# Simple monetary rules: many strengths and few weaknesses

**DOI:** 10.1007/s10657-020-09683-1

**Published:** 2021-01-12

**Authors:** John B. Taylor

**Affiliations:** Hoover Institution, Stanford University, Stanford, USA

**Keywords:** Rules, Discretion, Complexity, Monetary policy, Taylor rule, Zero-bound, K1, E1, E5

## Abstract

This paper endeavors to examine the basic idea in Richard Epstein’s book Simple Rules for a Complex World. It does so by considering a specific simple rule which was explicitly designed for complex world. A basic idea in Epstein’s book is that the more complex is the world the better is the case for simple rules. To show this, he develops six simple rules pertaining to the rights of individuals, first possession, contracts, torts, government eminent domain and the power of taxation to provide public goods. This paper considers one rule rather than six rules, and it looks at monetary policy rather than policy in general. While the context is different, the case for simple rules made here provides a useful comparison with the case made by Epstein.

## Introduction

In this paper I examine a fundamental idea in Richard Epstein’s book *Simple Rules for a Complex World,* namely, that the more complex is the world the better is the case for simple rules. I consider a specific simple rule which was explicitly designed for a complex world, and I focus on monetary issues rather than society in general. Nevertheless, the case for simple rules put forth here has important similarities with the case put forth by Epstein. The monetary rule is amazingly simple compared to the very complex economic world within which it was designed to operate. I show that this simplicity is a major strength, though some have argued that it is a weakness.

## Historical context

The idea of simple monetary rules goes back a long way in economics—since the formal beginnings of the subject. Adam Smith (1776) argued in *The Wealth of Nations* that “a well-regulated paper-money” could improve economic growth and stability in comparison with a pure commodity standard, as explained by Asso and Leeson (2012). Henry Thornton (1802) wrote in the early 1800s that a central bank should have the responsibility for price level stability and should make the mechanism explicit and “not be a matter of ongoing discretion,” as Robert Hetzel (1987) showed. David Ricardo (1824, pp. 10–11) wrote in his Plan for the Establishment of a National Bank that government ministers “could not be safely entrusted with the power of issuing paper money” and advanced the idea of a rule-guided central bank. Knut Wicksell (1907) and Irving Fisher (1920) in the early 1900s proposed policy rules for the interest rate or the money supply to avoid the kinds of monetary induced disturbances that led to hyperinflation or depression. Henry Simons (1936) and Milton Friedman (1948, 1960) continued in that tradition recognizing monetary policy rules—such as a constant growth rate rule for the money supply—would avoid such mistakes in contrast with discretion.

The goal of these proposals was a monetary system that prevented monetary shocks and cushioned the economy from other shocks, and thereby reduced the chances of inflation, financial crises, and recession. The idea was that a simple monetary rule could avoid monetary excesses whether due to government deficits, commodity discoveries, or mistakes by government. The choice, as discussed in Taylor and Williams (2011), was between simple rules and chaotic policy whether caused by exogenous shocks like gold discoveries or shortages.

## From a complex rule to a simple rule

The specific rule that I consider here describes the settings for the interest rate—a policy instrument, or a tool, of monetary policy, which is an important part of overall economic policy. The interest rate is the short-term rate—the federal funds rate, and it is set by an agency of government—the central bank—the Federal Reserve System in the United States. For concreteness, I focus on the so-called Taylor Rule because it is discussed widely in both academic and policymaking circles, and thus brings practical policy making into play.

The simple rule originally came about in a search for an optimal rule. The economic world within which the search for the optimal rule took place was a complex economy described by standard economic models with hundreds of variables and interrelationships over time. Thus, not surprisingly the optimal rule that came out of the original research was very complex. It had hundreds of variables driving the policy instrument including many lags to account for the intertemporal relationships.

The complexity led to serious doubts about the usefulness of the research. Some raised questions about whether the results would ever be applied in practice. Indeed, anyone with policy experience—which I had gained through two stints at the Council of Economic Advisers (CEA) in Washington—could see that the complex rules would not be useful. The monetary economist David Laidler who had been a proponent of rules argued that we were stuck with discretion forever; rules were so complex that they were essentially discretion. Allan Meltzer argued otherwise, however, and encouraged me to work more on the problem. He asked me to present a paper on the subject at a conference in 1992 in Pittsburgh.

The Taylor rule emerged from a policy evaluation project at Stanford. The question we had to ask was: Could a simple monetary policy rule be designed that could be responsibly recommended to policy makers in practice, but that was consistent with what research was telling us about the key properties of very complex optimal rules?

It turned out that the answer was yes. The most promising design had the interest rate—the federal funds rate—rather than money supply or the monetary base as the instrument on the left-hand side of the rule. The Fed was not even talking publicly about its settings for the federal funds rate back then, so there was a leap of faith and much initial criticism of that design. Discussions with Alan Greenspan, then Chair of the Fed, gave me a degree of confidence that this design was workable. I tested the waters publicly by writing the idea up in informal terms in the 1991 Economic Report of the President. We showed that the Fed’s interest rate settings could, in effect, be thought of as following a systematic policy, not a complex or discretionary “Greenspan standard” as many had argued at the time. In fact, Greenspan later joked that the Fed deserved an assist in the developing the Taylor rule.

In any case the research we were drawing on showed that the policy interest rate need only react, at least as an approximation, to a few variables. Amazingly only two. It should react if the *inflation rate* moved away from target and if *real GDP* moved away from its potential. Of course, we had to have an inflation target, and I decided that a reasonable inflation target was 2%. That later became the actual target for many central banks.

The research also showed that the interest rate reaction to inflation should be greater than 1, and 1.5 was a representative value: The interest rate would be raised by 1.5 percentage points if the inflation rate rose by 1 percentage point and it would be lowered by the same amount if inflation fell by 1 percentage point. The research also said that the interest rate should be lowered a bit if real GDP fell relative to potential; I chose the value of 0.5 so that the interest rate would fall by 0.5 percentage point if the gap between real GDP and potential fell by 1 percentage point. The research also showed that the interest rate should not react much to other variables, such as the exchange rate or other asset prices; this was a big factor in its simplicity.

Finally, we needed an equilibrium interest rate—a value for the interest rate when inflation equaled to its 2% target and real GDP equaled potential GDP. I chose 2% for the real rate based mostly on historical experience in the United States and noted some consistency with the real GDP trend growth rate of around 2% at the time. Of course, 2% real plus the inflation target at 2% implies a 4% nominal interest rate.

The bottom line in one short sentence: set the interest rate equal to 1.5 times the inflation rate, plus 0.5 times the GDP gap, plus 1. And, as I explained in the original paper, this was not a recommendation to follow a rule mechanically. Judgement is required to implement the rule. To quote from the paper, the objective was to “preserve the concept of such a policy rule in a policy environment where it is practically impossible to follow mechanically any particular algebraic formula that describes the policy rule.”

So, what I presented back in 1992 and what people started calling the Taylor rule was not the result of one short paper (Taylor (1993)) and a simple view of the world. It represented nearly 25 years of research work with large complex models. It was not a curve fitting exercise in which any old instrument of monetary policy was regressed on a bunch of variables and their lags. A regression over the past couple of decades at that time would not have yielded such a rule. I showed that the rule had similarities to the decisions taken by the Fed during Alan Greenspan’s term as Chairman thus far, but that was more to talk about deviations from the rule as in the stock market crash of 1987.

## The importance of simple rules

To this day people say that such rules are too simple because they omit certain variables. Well, they were simple, because they were made to be simple. At the time people were coming up with all sorts of complex rules that included many types of variables, including asset prices. These rules were too complex to be workable in practice. It was amazing that they could be simplified. Rules from which certain variables were removed gave just as good a performance in many models as more complex rules. Simple rules were nearly as good as optimal complex rules, and they were certainly something more practical for policy makers to work with.

Another advantage was that a simple rule is more robust than complex rules over a wide range of models and experiences. Levin and Williams (2003) and Orphanides and Williams (2008) found that more complex fully optimal policies performed poorly in some models, while simple rules performed well in a wide variety of models. Optimal policies can be overly fine-tuned to a specific model. That is fine if that model is correct, but not if it is incorrect. Simple monetary policy rules incorporated basic principles such as leaning against the wind of inflation and output. Because they were not fine-tuned to specific assumptions, they were more robust than other rules. Simulations show that the simple rule worked well by taking key regularities into account. People came up with more complex rules that include many types of variables, but removing certain variables gave better performance.

Yet another advantage of simple rules was that they overcame difficulties about how complex rules could be explained to people in practice. While judgement is required, anyone with policy experience could see that the complex rules would not be practical.

The simple rules that were suggested led in other directions which helped to reinforce their use. Economists learned that policy rules helped them explain unusual phenomena, such as the positive correlation between inflation surprises and exchange rate movements. Interest in simple policy rules also grew beyond academia and central banks: Wall Street economists found them to be useful rules of thumb for predicting central bank actions as explained by Lipsky (2012). Also, policy rules affected other equations in models because with them it became more reasonable to assume that “economic agents” develop their own rules of thumb when monetary policy becomes more predictable. And it enabled economists to consider policy robustness in a rigorous way, as emphasized by McCallum (1999) and continued by Wieland et al. (2016).

The approach was also applied internationally. Research with the models demonstrated the near global optimality of a simple global rule in which each central bank followed a simple rule for its own country assuming other central banks would do the same. Thus, the research showed that rules-based monetary policy would lead to good macroeconomic performance in the national economy and in the global economy. This in turn led to suggestions for designing a rule-based international monetary system based on policy rules in each country. (See Taylor (2016)).

That the simple rules appeared to work well in practice also helped to reinforce confidence in the rules that were being suggested. Central banks appeared to be moving toward more transparent rules-based policies in 1980s and 1990s, including through a focus on price stability, and economic performance improved. This connection between the rules-based policy and better performance was detected by Clarida et al. (2000). There was an especially dramatic improvement compared with the 1970s. Mervyn King (2003) called it the NICE period for non-inflationary consistently expansionary, and there was also a near internationally cooperative equilibrium (another NICE) among most developed countries as there were few complaints about spillovers. By the year 2000 many emerging market countries joined the rules-based policy approach. Their improved performance contributed to global stability.

There are several reasons why central banks liked the simple rules. These include:

1. Time inconsistency. The time inconsistency problem calls for the use of a policy rule in order to reduce the chance that the monetary policy­makers will change their policy after people in the private sector have taken their actions. See Taylor (1993) for an explanation.
2. Clearer explanations. If a policy rule is simple, it can make explaining monetary policy decisions to the public or to students of public policy much easier. It is difficult to explain why a specific interest rate is being chosen at a specific date without reference to a method or procedure such as would be described by a policy rule. The use of a policy rule can mean a better educated public and a more effective democracy. It can help to take some of the mystique out of monetary policy.
3. Less short-run political pressure. A simple policy rule is less subject to political pressure than complex or discretionary policy. If monetary policy appears to be run in an ad hoc and complicated way rather than a systematic way, then politicians may argue that they can be just as ad hoc and interfere with monetary policy decisions. A monetary policy rule which shows how the instruments of policy must be set in a large number of circumstances is less subject to political pressure every time conditions change.
4. Reduction in uncertainty. Simple policy rules reduce uncertainty by describing future policy actions clearly. The use of simple monetary policy rules by financial analysts as an aid in forecasting actual changes in the instruments would reduce uncertainty in the financial markets.
5. Teaching the art and science of central banking. Simple monetary policy rules are a good way to instruct new central bankers in the art and science of monetary policy. In fact, it is for exactly this reason that new central bankers frequently find such policy rules useful for assessing their decisions.
6. Greater accountability. Simple policy rules for the instrument settings allow for more accountability by policymakers. Because monetary policy works with a long and variable lag, it is difficult simply to look at inflation and many other variables and determine if policymakers are doing a good job.
7. A useful historical benchmark. Simple policy rules provide a useful baseline for historical comparisons. For example, if the interest rate was at a certain level at a time in the past with similar macroeconomic conditions to those of today, then that same level would be a good baseline from which to consider today’s policy actions.

## Complex rules and discretion: debate continues

Of course, there were and are arguments made against using simple policy rules. Often the arguments are not that complex rules should be used instead of simple rules in a complex world, but rather that no rule—that is, discretion—is needed in a complex world.

For example, Summers (2014) and I had a debate on the subject of rules versus discretion. Summers began by saying: “John Taylor and I have, it will not surprise you…a fundamental philosophical difference, and I would put it in this way. I think about my doctor. Which would I prefer: for my doctor’s advice, to be consistently predictable, or for my doctor’s advice to be responsive to the medical condition with which I present? Me, I’d rather have a doctor who most of the time didn’t tell me to take some stuff, and every once in a while said I needed to ingest some stuff into my body in response to the particular problem that I had. That would be a doctor who’s [advice], believe me, would be less predictable.”

Thus, Summers argues in favor of relying on an all-knowing expert, a doctor who does not perceive the need for, and does not use, a set of guidelines, but who once in a while in an unpredictable way says to ingest some stuff.

But as in economics, there has been progress in medicine over the years. And much progress has been due to doctors using checklists, as described by Gawande (2009). Of course, doctors need to exercise judgement in implementing checklists, but if they start winging it or skipping steps the patients usually suffer. Experience and empirical studies show that checklist-free medicine is wrought with dangers just as rules-free, strategy-free monetary policy.

In another development that questioned simple policy rules, Bernanke (2010) offered another definition of a rule. But the rule that Bernanke had in mind is not a rule in the sense used it in this paper, or that many others have used it.

Rather it is a concept that all you really need for effective policy making is a goal, such as an inflation target and an employment target. In medicine, it would be the goal of a healthy patient. The rest of policymaking is doing whatever you as an expert, or you as an expert with models, thinks needs to be done with the instruments. You do not need to articulate or describe a strategy, a decision rule, or a contingency plan for the instruments. If you want to hold the interest rate well below the rule-based strategy that worked well during the Great Moderation, as the Fed did in 2003–2005, then it’s ok, if you can justify it in terms of the goal.

Bernanke (2010) and others have argued that this approach is a form of “constrained discretion.” It is an appealing term, and it may be constraining discretion in some sense, but it is not inducing or encouraging a rule as the language would have you believe. Simply having a specific numerical goal or objective function is not a rule for the instruments of policy; it is not a strategy; in my view, it ends up being all tactics. I think there is evidence that relying solely on constrained discretion has not worked for monetary policy.

Another evolution of the policy rule concept is the idea of “forecast targeting” as developed by Svensson (1998) and Woodford (2012). Woodford entitled his 2012 paper “Forecast Targeting as a Monetary Policy Strategy,” emphasizing that this alternative approach is a strategy. There is a close connection between “inflation forecast targeting” and policy rules for the instruments. In Taylor (2012) I argued that they were the dual solution to the same problem, much like first-order conditions and decision rules provide dual and complementary answers to the same optimization problem. One can learn from both approaches.

According to this approach the central bank would choose its policy interest rate so that a linear combination of its forecast of different variables would fall along a given path. While an interest rate path can be calculated using this approach it need not yield a simple policy rule for the instruments. The central bank would have the job of deciding on the instrument setting, and this might cause tension with some of the reasons for policy rules given above.

I am frequently asked how a system of policy rules can work in practice when politicians and government officials are so often called on to “do something, anything,” and feel strong pressure to do so. Rules sound good, skeptics say, but rules mean you do nothing, and that is impossible in today’s charged political climate and hour to hour or even minute to minute news cycle. My colleague George Shultz describes the problem as “the urge to intervene” when he describes events where, as a policy maker, he resisted that urge.

But simple rules for monetary policy do not mean that the central bank does not take action to change the instruments of policy (interest rates or the money supply) in response to events, or to provide loans in the case of a bank run. Rather it means that they take such actions in a predictable manner. And inaction can mean that one has deviated from a rule or a strategy. A decision by government regulators, for example, not to act when financial institutions take on risk beyond the limits of the rules and regulations is inaction and certainly is not observing the rule of law. It is important for policymakers to be able to explain that a policy strategy involves a series of actions.

Some claim that crises force policy makers to deviate from rules. This was the case in the Global Financial Crisis of 2008 and in the Coronavirus Crisis of 2020 where many called for special actions and deviations from rules. But a crisis may be the worst time to deviate from rules. In a crisis, increased clarity about the strategy rather than increased unpredictability is needed. This was clear following the bailout\s of 2008 when few knew what to expect the next time because there was no strategy. So, the crisis got worse. The sooner people can make decisions with knowledge of the rules, the sooner will be the recovery.

## Simple rules in practice

Has the research on simple policy rules directly affected the analysis and decisions of monetary policy makers and their committees? The question is difficult to answer, though increased central bank transparency is aiding investigations.

Kahn (2012) provided much useful detail about how simple policy rules have been the subject of discussion at the Federal Reserve, using transcripts and records of the Federal Open Market Committee (FOMC) meetings starting in the 1990s. He also considers the proceedings at other central banks, including the ECB, the Bank of Japan and the Bank of England. There was a great deal of discussion related to policy rules through the 1990s. This corresponds to the time-period when actual policy decisions were rule-like. There was also much mention of simple policy rules in the deliberations at other central banks during the Great Moderation period. I have used records of deliberations at the Norges Bank to assess the contagion of deviations from policy rules (the degree to which central banks follow each other) in recent years. I have also benefited from informal discussion with many central bankers in other countries over the years, and I found that they are all familiar with policy rules and understand their value.

An important research question is how discussions of policy rules evolved recently at the FOMC, especially during the period in 2003–2005 when there was a deviation from policy rules. To be sure, the records of the meetings and discussions may miss informal conversations and other key elements of any decision process at central banks, so some “investigative reporting” may be needed. Mallaby (2016) writes about the FOMC decision to keep interest rates low and to say that they would be low “for a considerable period,” and does not indicate one way or the other whether there was discussion that the rate was too low based on policy rules. Later, Bernanke (2010) argued that they were not too low based on policy rules if one used forecasts of inflation rather than actual inflation. As I pointed out in Taylor (2010), however, the Fed’s forecasts were lower than actual inflation at the time, and the forecast turned out to undershoot inflation over the forecast horizon.

Much of the policy changes in the 2009–2013 period were “balance sheet” operations as the Fed purchased Treasury securities and mortgage-backed securities in large-scale. Such actions again occurred in the Coronavirus Crisis of 2020. It is difficult to classify these actions as rule-like in the sense I have used the term in this paper.

However, the normalization process designed and described in the Fed’s 2014 “Policy Normalization Principles and Plans” is consistent with a more rules-like approach, in which the FOMC “intends to reduce the Federal Reserve's securities holdings in a gradual and predictable manner….” The “Addendum to the Policy Normalization Principles and Plans” provided useful details about how the FOMC intended to gradually reduce the Fed's securities holdings by decreasing its reinvestment of principal payments to the extent that they exceed gradually rising caps.

Janet Yellen’s (2017a, b) discussions of monetary policy rules also broke ground in describing how policy rules were again being used at the Federal Reserve. A follow-up presentation by Stanley Fischer (2017) and a new section of the June 2017 Monetary Policy Report continue in this vein.

Yellen (2017a) summarized the Fed’s strategy for the policy instruments, saying that “When the economy is weak and unemployment is on the rise, we encourage spending and investing by pushing short-term interest rates lower…. Similarly, when the economy is threatening to push inflation too high down the road, we increase interest rates to keep the economy on a sustainable path and lean against its tendency to boom and then bust.” She then described “price stability” as a level of inflation of “2 percent a year.” One could certainly add more detail, but the statement includes the signs of the responses by the policy instruments, though not the magnitudes. It mentions key factors driving the responses. And it gives numerical values for three key parameters.

Yellen (2017b) also provided charts and references to the specific policy rules. The purpose was to compare actual Fed policy with the Taylor rule and other rules, and then explain any differences. I think people found that useful, and it was good to see clarification of how the FOMC uses such policy rules in a constructive manner. An algebraic way to summarize the words in the presentations would be: *r* = *p* + *ay* + *b*(*p* – 2) + 1 with *a* > 0 and *b* > 0, where *r* is the federal funds rate, *p* is the rate of inflation, and *y* = 2.3(4.75-*u*) where *u* is the unemployment rate. (The 2.3 comes from Yellen (2012)). In contrast the Taylor rule is *r* = *p* + 0.5*y* + 0.5(*p* – 2) + 2. This clearly provided context for a candid discussion. Stanley Fischer (2017) gave a follow-up talk which takes a similar approach; he referred to decisions made in 2011 and more generally, explained how the rules-based analysis feeds into the discussions and is evaluated by the FOMC to arrive at a policy decision.

Federal Reserve Board’s semi-annual Monetary Policy Report also started in (2017) to contain a whole new section called “Monetary Policy Rules and Their Role in the Federal Reserve’s Policy Process.” The section lists three “key principles of good monetary policy” that the Fed says are incorporated into policy rules; it then lists five policy rules, including the Taylor rule and four variations on that rule that the Fed uses, with helpful references in notes.

The three principles sound quite reasonable: For one of them, sometimes called the “Taylor Principle,” the Fed is quite specific in that it gives the numerical range for the response of the federal funds rate to the inflation rate.

More information, including some algebra, is given in the 2017 Report. One of the five policy rules, which the Fed calls the “Taylor (1993) rule, adjusted,” is based on the Reifschneider and Williams (2000) paper on the zero-lower-bound. The Fed describes these rules using the unemployment rate rather than real GDP, relying on Okun’s Law, the empirical connection between the real GDP/potential GDP gap and the unemployment rate. One of the rules, what the Fed calls the “balanced-approach rule” is the Taylor rule with a different coefficient on the cyclical variable.

The 2017 Report compared the FOMC’s settings for the federal funds rate with policy rules. It showed that the interest rate was too low for too long in the 2003–2005 period according to the Taylor rule, and that, according to three of the rules, the current fed funds rate should be moving up. The Fed makes these calculations using its estimate of time varying neutral rate of interest. Aside from being positive about the three principles, it did not say much about its own policy strategy in the document.

The Report focused on differences, rather than similarities, in the policy rules, and on the differences in inputs to the policy rules. The differences in measures of inflation, the equilibrium neutral interest rate, and other variables are part of monetary policy making and always will be. They are a reason to use policy rules as a means of translating these differences in measurement into differences about policy in a systematic way.

It is also worth noting that there are signs that the concept of policy rules is affecting practical thinking on the international front. Paul Volcker (2014) argues that “the absence of an official, rules-based, cooperatively managed monetary system has not been a great success.” Raghu Rajan (2016) writes that “what we need are monetary rules that prevent a central bank’s domestic mandate from trumping a country’s international responsibility.” And Mario Draghi (2016) states that “We would all clearly benefit from…improving communication over our reaction functions…” All are suggesting a more rule-based approach to the international monetary system.

## Recent revival of simple rules

**Fig. 1:**
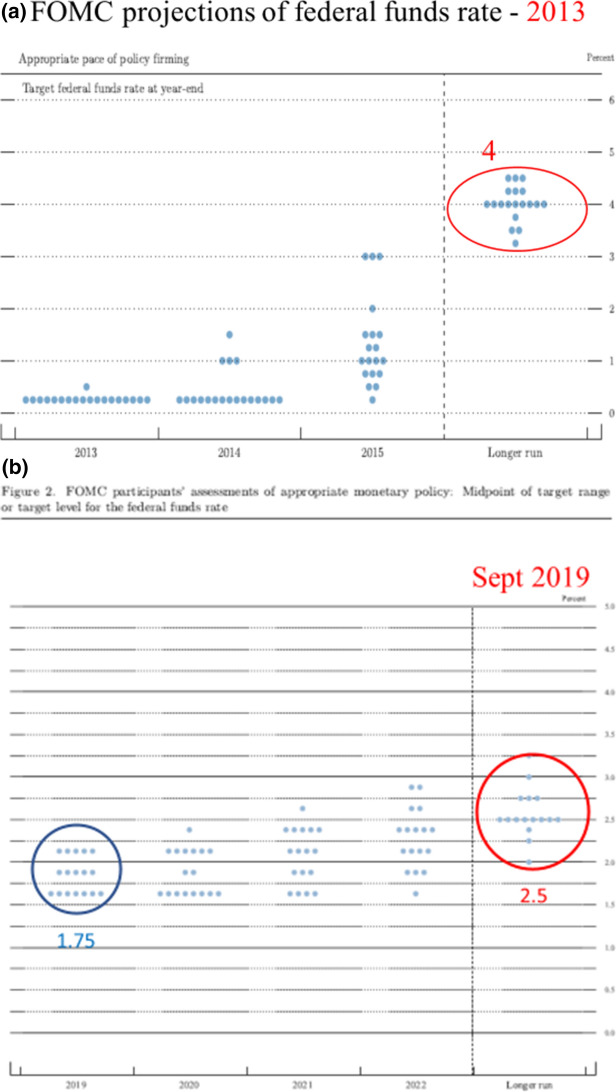
**a** FOMC participants’ assessments of appropriate interest rate given by midpoint of target range or target level for the federal funds rate in December 18, 2013. **b** FOMC participants’ assessments of appropriate interest rate given by midpoint of target range or target level for the federal funds rate on September 18, 2019

In the years 2017–2019 research on the economic impact of different monetary policy rules appears to be enjoying a revival. For one thing the decisions of the Fed moved in this direction as shown by the FOMC’s “dot-plots” in Fig. 1a, b. The interest rate moved up from near zero in a positive direction during this period.

At a monetary policy conference on strategies for monetary policy held at the Hoover Institution at Stanford University, Mertens and Williams (2020) evaluated different monetary policy rules using a new Keynesian model and presented the results. At a monetary policy conference held at the Federal Reserve Bank of Chicago in June 2019, Sims and Wu (2019) evaluated different monetary policy rules using a new calibrated structural model and presented the results. And recently, Bernanke et al. (2019) examined ten different monetary policy rules using the Fed’s FRB/US model.

**Fig. 2:**
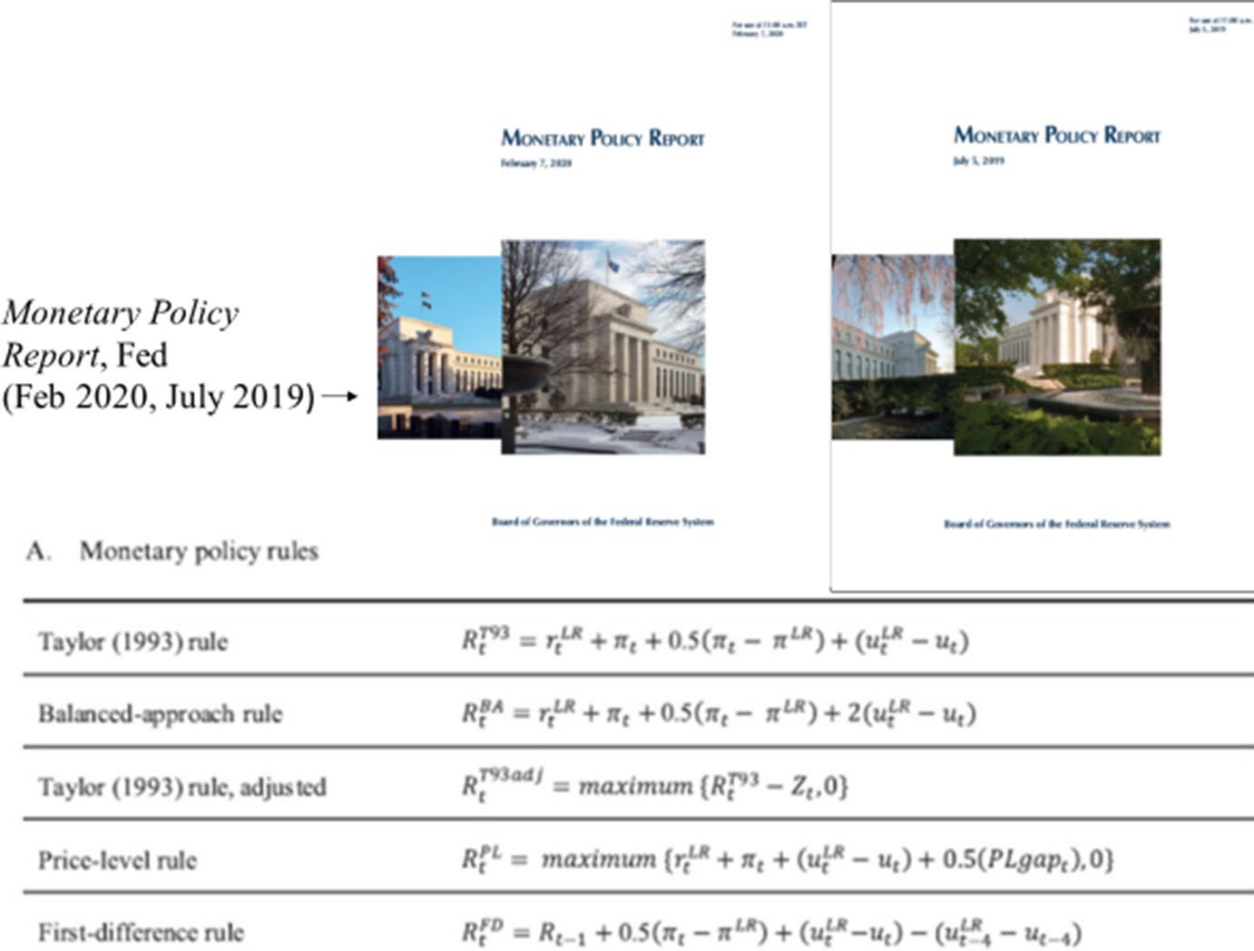
*Monetary Policy Report*, Federal Reserve (February 2020 and July 2019)

The new section on monetary policy rules in the Fed’s twice-per-year Monetary Policy Reports (2017,2018a,b,^2019^,^2020^) continued. See Fig. 2. The five different policy rules are being presented and compared with actual policy. Cochrane et al. (2019) evaluated the monetary policy rules presented in the Fed’s Monetary Policy Report at the Hoover Institution monetary conference as did Eberly et al. (2019) at the Chicago Fed monetary conference.

The monetary policy rules or strategies in these studies are stated in terms of the instruments of policy, such as the federal funds rate. It is of significance that the focus has been on instrument rules rather than forecast targeting rules. Another recent example of research on simple instrument rules is the work by Belognia and Ireland (2019) in which the money supply, rather than the federal funds rate, is the instrument.

Of course, monetary policy rules were the subject of much quantitative research by monetary economists at the Fed and elsewhere in the 1970, 1980s and 1990s, but that had diminished in the past 15 years or so. So, what explains the current revival?

Part of the explanation is simply revealed preference: A recent survey of monetary economists, including former Fed officials and other Fed watchers, conducted by Cecchetti and Schoenholtz (2019), reveals a desire for more and better descriptions of policy rules or reaction functions for the instruments. This corresponds to statements by central bank leaders mentioned above and the more recent statement by the current Chair of the Federal Reserve Board, Jerome Powell (2018), who said that “I find these rule prescriptions helpful”.

**Fig. 3:**
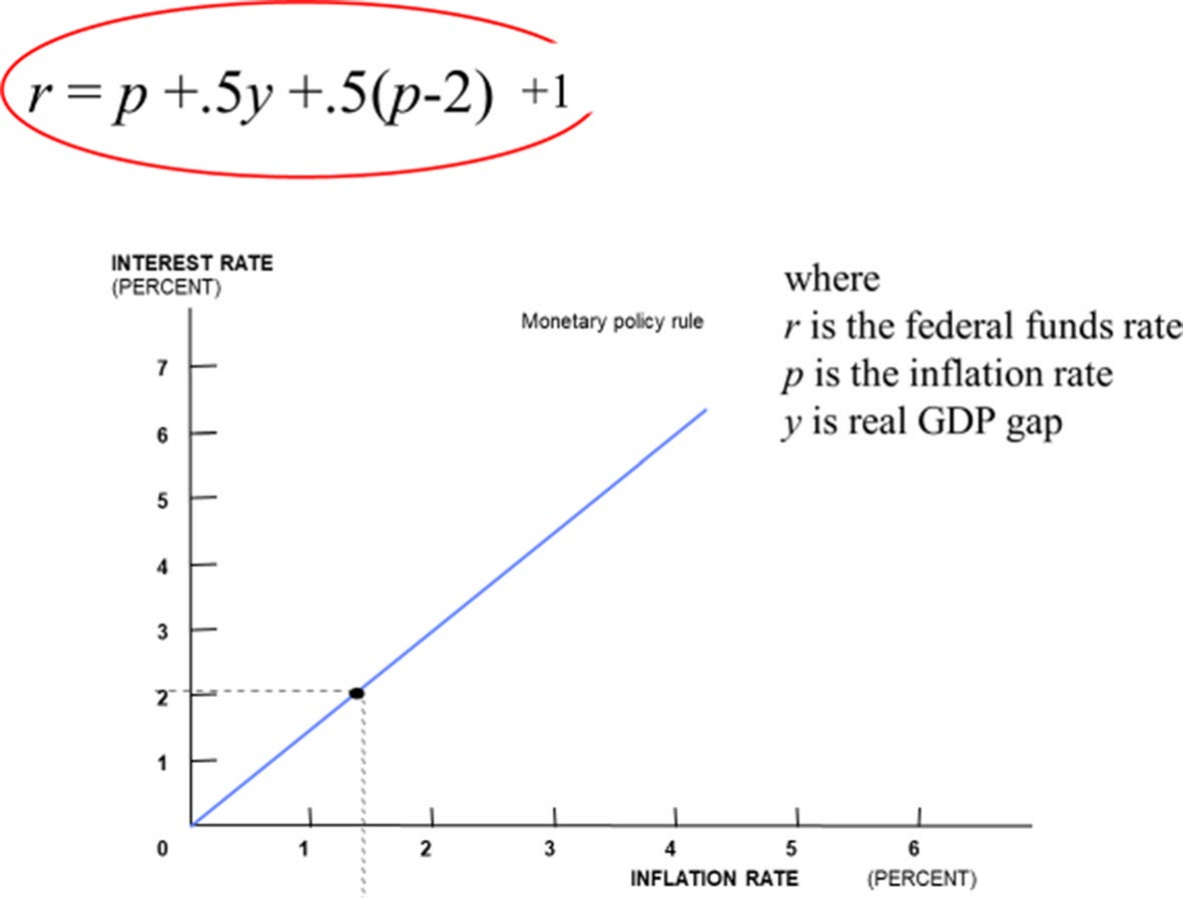
Taylor rule with an equilibrium real interest rate of 1 percent rather than 2 percent. In this chart, the GDP gap (y) is assumed to be − 0.5 percent

Another explanation for the renewed research on simple policy rules is the need to improve monetary policy in light of recent developments. For example, an estimated decline in the equilibrium real interest rate as studied by Laubach and Williams (2016) has created a greater concern about policy hitting the effective lower bound on the interest rate. Figure 3 shows that one way to deal with this is simply to lower the intercept in the policy rule.

But if the equilibrium rate is even lower, this may call for a different type pf policy rule to deal with the lower bound in some way, and therefore for proposals for designing such policy rules and an evaluation of the effectiveness of such rules. This motivation clearly underlies the work on negative interest rates by Lilley and Rogoff (2020) and Bordo and Levin (2019) on monetary policy rules.

Moreover, there were disappointments about monetary policy performance going into the great recession, and some of the blame is placed on deviating from rules. There are also concerns about excessive exchange rate volatility and big swings in international capital flows which indicate the need for an international monetary reform based on rules-based policy. In addition, there is a recognition that rules are helpful, even necessary, to evaluate the effect of unconventional monetary instruments. This view was expressed by Sims and Wu (2019) at the Chicago Fed conference and by Brian Sack former vice president at the New York Fed.

Gagnon and Sack (2018) similarly argue that “it would make sense for the policy rule governing asset purchases to be similar in nature to the policy rule that describes the policy rate. However, that has not been the case so far in the United States. To determine a policy rule that is “similar in nature requires specifying the nature of QE effects and comparing them to the effects from the federal funds rate….

There are two important features about the revival of work on simple policy rules. First the interest rate rules examined embody the “greater-than-one’ or Taylor principle in which the coefficient on inflation in the policy is greater than one. The rules in the Fed’s Monetary Policy Reports have this property, and Reports note that “Policy rules can incorporate key principles of good monetary policy. One key principle is … that, to stabilize inflation, the policy rate should be adjusted by more than one-for-one in response to persistent increases or decreases in inflation.”

Second, very few of these studies have described the policy rule in terms of instrument of quantitative easing, namely large-scale asset purchases under quantitative easing. One exception is the policy rule studied by Sims and Wu (2019), which proposes an equation similar to the Taylor rule but for purchases of securities rather than for the interest rate. Eberly et al. (2019) assumed that the instrument is the slope between the federal funds rate and the10-year Treasury rate, but they offer no quantitative model of how actions with the instruments affect the slope. Perhaps this is due to the ongoing controversy about the impact of quantitative easing as exemplified by differences between the papers by Hamilton (2019), Greenlaw et al. (2018), and Bordo and Levin (2019), who find no significant effects and Gagnon (2019) who does. Bordo and Levin, for example, report that “Our empirical analysis indicates that QE3 was not an effective form of monetary stimulus and that unconventional monetary policies in the Eurozone and in Japan have proven to be similarly ineffectual.”

## Conclusion

This paper has shown how simple policy rules emerged as a guide to monetary policy in an increasingly complex world. At first there was no workable alternative: Complex models seemed to imply complex rules on the one hand or no rules on the other. But research eventually showed that simple rules were good alternatives in theory and in practice, and so simple rules such as the Taylor rule began to be discussed and used. Soon more advantages of simple rules became apparent.

There is much evidence that that simple rules affected actual monetary policy in the 1980s and 1990s. But deviations sometimes arose as in the period before the Global Financial Crisis and in the Coronavirus Crisis. Nevertheless, interest in simple rules at central banks has continued for many reasons cited in the paper. The debate continues, however, and more progress is still needed especially in international monetary policy. The theoretical, empirical and historical experience examined in this paper sheds light on the important topic of this conference on the strengths and weaknesses of simple rules.
